# Prevalence and family structures related factors associated with crown trauma in school children resident in suburban Nigeria

**DOI:** 10.1186/s12903-016-0314-9

**Published:** 2016-11-05

**Authors:** T. A. Oyedele, A. T. Jegede, M. O. Folayan

**Affiliations:** 1 Department of Surgery, Ben Carson School of Medicine, Babcock University, Ilisan-Remo, Ogun Nigeria; 2 Dental Department, Babcock University Teaching Hospital, Ilisan-Remo, Ogun Nigeria; 3 Department of Child Dental Health, Faculty of Dentistry, Obafemi Awolowo University, Ile-Ife, Osun Nigeria

**Keywords:** Traumatic dental injuries, Socioeconomic status, Parenting, Gender

## Abstract

**Background:**

Multiple risk factors have been identified for traumatic dental injuries, including crown fractures, in various age groups and various populations. The objective of this study was to determine the prevalence, risk factors and family related factors for crown trauma among 8 to 16 year-old children in a suburban population, Nigeria.

**Methods:**

This is a secondary analysis of a data of 2107 children collected through a school-based survey. Study participants were 8 to 16 year-old resident in suburban Nigeria. The independent variables for the study were age, sex, socioeconomic status, and birth rank, family size and parenting status (one parent, step parent, both parents, and guardians). Details were collected using an interviewer administered questionnaire. Intraoral examination was conducted to identify presence of crown fracture. The independent variables associated with and predictors of crown trauma were determinedusing chi-square and logistic regression analysis respectively.

**Results:**

Only 167 (7.9 %) of the 2107 study participants had crown trauma. The teeth level prevalence of crown trauma was 0.33 %. Children with middle socioeconomic status had reduced odds of having crown trauma when compared with children with low socioeconomic status (AOR 0.50; CI 0.32–0.80). The odds of having crown traumawas more than doubled in males when compared with females (AOR 2.41; CI 1.72–3.39) and almost doubled in children living with single parents when compared with children living with both parents (AOR 1.94; CI 1.29–3.05).

**Conclusions:**

The prevalence of crown traumawas low in this study population. Being a female and having lowsocioeconomic status significantly reduced the risk factors for crown traumawhile living with single parents increased risk for crown trauma.

## Background

Traumatic dental injuries (TDI) are injuries to the oral cavity that often occurs in childhood and adolescence [1]. It comprises about 5 % of all injuries for which people seek treatment [1, 2]. It can cause irreversible dental loss and complications several years after initial injury or treatment [3]. Also, the negative impact traumatic dental injury has on the quality of life of affected individuals, the associate costs of treatment and the psychological impact resulting from associated morbidity makes TDI a serious public dental problem in children [3–7] and a research issue.

The prevalence of TDI range from 3.9 to 58.6 % [2] with variations noticed between countries and between populations. Many studies have reported a higher prevalence of TDI for males due to more male involvement in accident prone events such as aggressive plays, contact sports, and accident prone adventures [8, 9]. The prevalence of TDI is however, on the increase in females due to their increasing participation in contact sports [2, 10, 11]. The most common aetiology of TDI is falls [12, 13], followed by accidents, violence and sports [14–16].

There are conflicting reports on the association between the prevalence of TDI and socioeconomic status [17–20]. While some studies report that the prevalence of TDI is highest in children with high socioeconomic status [18, 21], others have reported higher prevalence of TDI in children with low socioeconomic status [22, 23]. These findings may point to possible differences in the culturisation of different populations. Frujeri et al. [24] reported that while the children with high socioeconomic status have access to leisure goods and equipment which may increase trauma occurrence, children with low socioeconomic status are more exposed to public area with little or no protective facilities equally increasing their risk for TDI like children with high socioeconomic status. Other possible risk factors for TDI include family structure related factors. Matthias et al. [25] reported higher prevalence of TDI in the first born. However, the likelihood of having a second TDI was higher in the last born [25]. Children from single parents also have higher risk for having TDI [23]. The most common type of TDI is simple crown fracture of the maxillary central incisors [26–28] irrespective of the type of classifications [29–31].

Various studies in Nigeria had tried to determine the prevalence of TDI and its association with age, sex and aetiological factors [12, 32–35]. These studies showed that TDI was more common amongst adolescents, males, and fall was the commonest aetiological factor [32–35]. None of these studies had however explored the role of family related variables as risk factors for TDI. This study is an effort to address this gapin knowledge. We therefore explored the risk factors associated with TDI in school children in Nigeria. Specifically, we investigate the association between age, sex, socioeconomic status, birth rank, family related factors and crown trauma, a proxy index for TDI, in school children resident in Ile-Ife, a suburban region in Nigeria.

## Methods

### Study population

This was a secondary data analysis. The primary data was generated for of a cross-sectional study that recruited 2107 children aged 8 to 16 years resident in Ife Central Local Government of Ile-Ife, a sub-urban town in the South-Western Nigeria. The objective of the study was to determine the co-morbidities associated with molar-incisor hypomineralisation in the population. The full details of the study, including the study methodology, had been reported by Oyedele et al. [36].

### Sampling technique

Study participants were selected through a multi-staged sampling technique. Selection of study participants through a proportional representation of public and private schools in the sampling frame helped to ensure that children from all the socioeconomic strata were recruited for the study. Enrolment into either a public or private school have been shown to be a sensitive procedure for distinguishing children from different socioeconomic status in the study environment: children with low socioeconomic status often attend the public school while those with middle and high socioeconomic status attend private schools [37, 38]. Children with high socioeconomic status attend private schools paying higher school fees when compared with those from the middle socioeconomic status [37].

First, Ife Central Local Government was divided into three geographical areas each consisting of four political wards. One political ward was selected from each of the geographical areas by ballot. Second, in each ward, the schools were stratified into public primary, private primary, public junior secondary, private junior secondary, public senior secondary and private senior secondary schools respectively. A list of schools obtained from the Osun State Ministry of Education was used for the stratification. One school was randomly selected from each stratum by balloting. In effect, six schools were randomly selected from each ward and 18 schools from Ife Central Local Government were enlisted for the study. Third, the lists of the children in each class in each of the selected schools were reviewed. Classes with the high numbers of children who met the age criteria for the study were selected for study participation. All selected study participants were invited to participate in the study and were given informed consent forms for their parents.

### Sample size

The sample size was determined by the statistical formula proposed by Araoye [39] using an estimated proportion of children with TDI of 9.1 % [34], a standard normal deviate set at 1.96 corresponding to 95 % confidence interval, and amargin of error of 0.05. The sample size required to determine the prevalence of traumatic dental injury for this, inclusive of a 10 % attrition rate, was 140 children. However, we had access to the data of 2107 thereby making the data robust enough to address the study objective.

### Data collection

The data collection tool captured details of the age, sex and socioeconomic status of each child. Age was the age at last birthday. The sex was determined using biological variables (male and female). The socioeconomic status of each child was determined through a multiple-item scoring index [40] developed from information about the mother’s level of education and the father’s occupation. The index had been used in prior studies in Nigeria [41, 42]. Each child’s social class was classified as class I (upper class), class II (upper middle class), class III (middle class), class IV (lower middle class), or class V (lower class). Respondents were also asked about the number of siblings they had, their birth rank and theparenting status. The parenting status was pre-classified as one parent, stepparent, both parents, and guardians. Respondents had to choose a parenting status that best represents their family structureduring the data collection process. Only children who returned the signed informed consent form from their parent/legal guardian and who assented to participate in the study were administered the questionnaire.

### Clinical examination

Each child was examined by one of the authors (TAO) under natural light, while seated on the school chair. The teeth were examined wet. However, debris was removed with a gauze swab where present. A trained assistant helped to record findings during intra-oral examination.

#### Crown trauma

Trauma to the anterior teeth of each study participant was determined using Ellis and Davey classification [31]. Trauma was classified as present when there was a simple fracture of the crown involving little or no dentine (Class I); extensive fracture of the crown involving considerable dentine (Class II) and exposure of dental pulp (Class III), or loss of the entire crown (Class IV). Visual assessment of tooth discoloration was also performed. Teeth, which appeared pink, yellow, dark grey, greyish-blue, greyish black or dark-brown were classified as discolouration arising from dental trauma. Tooth sensibility tests were not performed on the field.

### Intra-examiner reliability

Prior to the commencement of the study, TAO underwent a series of calibration exercises for the identification of TDIs and crown discolouration. Calibration for TDI was done using a coloured picture chart of TDI-affected teeth with different trauma classes. This was then followed by the examination and diagnosis of crown fracture and crown discolouration, in live patients at different intervals. The data were then subjected to unweight kappa scores analysis, to determine intra-examiner reliability. The intra-examiner reproducibility score for TDI was 0.90.

### Data analysis

The socio-economic status of the children was grouped into 3 during data entry. Classes1 and II were classified as high socio-economic status, class III remained the middle socio-economic status and classes IV and V were classified as low socio-economic status. Study participants were also grouped into three based on age: 8 to 10 years old, 11 to 13 years old; and 14 to 16 years old. Birth rank was grouped into first born, last born and other birth ranks. The number of sibling was grouped into 0–1, 2–4 and more than 4. Four was chosen as a reference point since Nigerian government has recommended that families limit themselves to four children [43]. The number of siblings was dichotomised for the logistic regression analysis: the two groups were one or no siblings and more than one sibling. Association between age, sex, socioeconomic status, birth rank, number of sibling and parenting status was determined using chi-square analysis. The predictors for presence of crown trauma were determined using logistic regression. The reference groups in the model were females, children from the low socioeconomic status, other birth ranks, more than one child and living with both parents. Factors whose *p* value was <0.40 during the tests of association entered into the model. Statistical analysis was conducted with the use of STATA version 11.0. Statistical significance was established at *p* values equal to or less than 0.05.

### Ethical consideration

Ethics approval for the study was obtained from the Obafemi Awolowo University Teaching Hospitals Complex, Ile-Ife Health Research Ethics Committee (ERC/2011/06/03). Approval was also obtained from the Ministry of Education and the Heads of all the schools that participated in the study. Only children whose legal guardian consented to their participation and those who gave assent to study participation were eligible to participate in the study.

## Results

The response rate for the study was 89 % Table 1 highlights the distribution of study participants by age, sex and socioeconomic status. The study participants comprised of 1125 (53.4 %) females and 982 (46.6 %) males. Of these, 167 (7.9 %) participants had crown trauma. Prevalence of TDI across age group shows 6.0, 9.8 and 7.3 % among 8–10 year-old, 11–13 year-old school children respectively. Also 197 (0.33 %) of the 58,996 teeth examined had crown trauma. More males than females had crown trauma (66.5 % vs 33.5 %; *p* ≤ 0.001). Also, more children in the age group 11–13 years (*p* = 0.04), and more children from the low socioeconomic class (*p* = 0.01) had crown trauma.

**Table 1:** Logistic regressions determining the odds of having TDIs in study Participants resident in Ile-Ife, Nigeria (*N* = 2107)

Variables	Crown trauma		*X* ^2^ (*P*-value)	Adjusted OR	95 % C.I	*P*-value
	Present *N* = 167 n (%)	Absent *N* = 1,940 n (%)				
Age						
8–10 years	28 (16.7)	441 (22.7)	6.52 (0.04)	1	-	-
11–13 years	75 (44.9)	691 (35.6)		0.55	0.35–0.87	0.01
14–16 years	64 (38.4)	808 (41.6)		0.73	0.45–1.16	0.18
Sex						
Female	56 (33.5)	1,069 (55.1)	28.7 (≤0.001)	1	-	-
Male	111 (66.5)	871 (44.9)		2.41	1.72–3.39	≤0.001
Socioeconomic status						
High	46 (27.5)	568 (29.3)	9.03 (0.01)	1	-	-
Middle	24 (14.4)	450 (23.2)		0.50	0.32–0.80	0.045
Low	97 (58.1)	922 (47.5)		0.87	0.60–1.27	0.58
Birth rank						
First born	58 (34.7)	626 (32.3)	2.32 (0.31)	1	-	-
Last born	31 (18.6)	461 (23.8)		1.29	0.83–2.02	0.26
Others	78 (46.7)	853 (43.9)		1.03	0.72–1.48	0.88
Number of siblings						
0–1	5 (3.0)	32 (1.6)	2.08 (0.35)	1	-	-
2–4	105 (62.9)	1,287 (66.3)		1.64	0.61–4.41	0.33
>4	57 (34.1)	621 (32.1)		1.52	0.54–4.27	0.43
Parental status						
Both parents	121 (72.5)	1,585 (81.7)	12.7 (0.01)	1	-	-
Single parent	31 (18.6)	192 (9.9)		1.98	1.29–3.05	0.002
Living with others	15 (8.9)	163 (8.4)		0.94	0.52–1.71	0.83

Table 1 also highlights the distribution of study participants by birth rank, number of siblings and family structure. There was no significant difference in the proportion of study participants who had crown trauma by birth rank (*p* = 0.31) and by number of siblings (*p* = 0.35). There was however a significant association between parenting status and presence of crown trauma (*p* = 0.01): more children living with single parents had crown trauma when compared with those who lived with both parents, stepparents and guardians.

In addition Table 1 shows the outcome of the logistic regression determining the predictors of crown traumaand their effect size. Three predictors of crown trauma were identified: socioeconomic class, gender and parental status. Children from the middle socioeconomic class were 50 % less likely to have crown trauma when compared with children from low socioeconomic class (AOR: 0.50; 95 % CI: 0.32–0.80; *P* = 0.045). Males had a 2.4 fold increased risk for crown trauma when compared with than females (AOR: 2.41; 95 % CI: 1.72–3.39; *P* ≤ 0.001). Also, children living with a single parent were two times more likely to have crown trauma when compared with children living with both parents (AOR: 1.98; 95 % CI: 1.29–3.05; *P* = 0.002).

Table 2 shows the pattern of crown trauma observed. The majority of crown trauma was Class I (77.2 %). Tooth discolouration was observed in only one (0.6 %) of the study participants.

**Table 2:** Classification of crown trauma observed in study participants resident in Ile-Ife, Nigeria (*N* = 167)

Tissue involved	Number of children (%)
Enamel only	129 (77.2)
Enamel and dentine	32 (19.2)
Enamel, dentine and pulp	3 (1.8)
Crown en-mass	3 (1.8)
Discolouration	1 (0.6)
Total	167 (100.0)

Table 3 shows the distribution of teeth affected by crown trauma. The upper left central incisors were the teeth most affected (44.2 %) followed by upper right central incisor (42.6 %). The least affected teeth were upper right canine (0.5 %), upper left canine (0.5 %), upper left first premolars (0.5 %) and lateral incisor (0.5 %). There were more teeth affected by TDI in the upper than lower jaw (*p* ≤ 0.001).

**Table 3:** Distribution of teeth in persons affected by crown trauma in children resident in Ile-Ife, Nigeria (*N* = 197)

Teeth affected	Number (%)
11	84 (42.6)
12	8 (4.1)
13	1 (0.5)
21	87 (44.2)
22	7 (3.6)
23	1 (0.5)
24	1 (0.5)
31	4 (2.0)
32	1 (0.5)
Total	197 (100.0)

## Discussion

This objective of the study was to determine factors associated with a crown fracture in school children age 8 to 16 in Nigeria. Specifically, we tried to identify risk indicators for crown trauma in the study population. The three significant risk indicators for crown trauma identified for the study population were being male, living with single parents and having high and low socioeconomic status. The child’s birth rank and the number of sibling the child had were not significant risk indicators for crown trauma.

Our study findings were similar to that of other studies in many respects. Like previous studies [8, 9], we found that males had increased likelihood of having TDI. The semi-urban nature of the study environment and the genderisation of roles in a highly patriarchal society like Nigeria increase the likelihood of females being more docile than males, and females being less engaged in contact sports and risky adventures. This observed gender differences in the proportion of male and females with TDI is changing in many communities in developed countries where more females are participating in contact sports [1, 10]. However, when we compare our study report to that of Adekoya-Sofowora et al. [34] who conducted a similar study in 13 to 15 year old children in Ile-Ife 7 years prior to this study, we did not notice any significant difference in the proportion of male and females who have TDI in the two studies despite the time difference. There may therefore be significant cultural differences in the domestication process of males and females in different cultures and environment leading to differences in ways males and females engage with their environment; and the impact this has on the tendency for children to sustain dental injuries.

Like Frujeri et al. [24], we also observed that there was no significant difference in the likelihood of children with high socioeconomic status having TDI when compared with children with low socioeconomic status. However, children with middle socioeconomic status had significant lower risk of having TDI when compared with children with low socioeconomic status. We agree with Frujeri et al. [24] postulation that while the children with high socioeconomic status have access to leisure goods and equipment that may increase trauma occurrence, children with low socioeconomic status are more exposed to public area with little or no protective facilities equally increasing their risk for TDI like children with high socioeconomic status. We think that our findings may imply that children with middle socioeconomic status may be less exposed to public area with little or no protective facilities when compared with children with low socioeconomic status, and may not have access to leisure goods and equipment which may increase trauma occurrence. In these ways, the child’s middle socio-economic status served as a means of reducing their risk for TDI.

Also like prior studies, the parenting status of the child can be a risk factor for TDI [44] just like it is for caries [45] and oral hygiene [46]: children living with single parents or with other people have significant higher occurrence of TDI when compared to children who live with both parents [44]. The pre-occupation of single parents with trying to provide for the financial needs of the family is often associated with neglect of small details about health care for children, especially oral health care [47].

Again, we observed that the upper central incisors were most often affected by TDIs like other studies [35, 48, 49], and the most common type of TDI is fracture involving the enamel only like others [2, 50]. The upper anterior teeth are more often involved in TDI when compared to the lower anterior teeth because the upper jaw is fixed to the skull making it rigid and increasing the impact of forces directed at the face more on the upper anterior teeth [51].

While the findings of this study do not differ significantly from findings of prior studies, the study was important as we were able to identify risk factors for crown fracture in the study population that had not been identified prior to this study. We were able to generate evidence of the association between the socioeconomic status of the child, the child’s parenting status and the risk of crown fracture. We were also able to show that the prevalence of TDI in the study environment appears to have remained constant over the last decade. This has implications for making plans and designing programmes to further reduce the incidence of crown fracture in the study population. School authorities will need to be aware of the need to provide and instil the use of protective headgears by students who engage in contact sports. Eigbobo et al. [52] showed clearly that one high risk public area for TDI in Nigeria are the public schools where the playgrounds are free of grass and often made of laterites. While there is very little that can be done to change the biological factors this study has identified as risk factors for crown fracture – gender, socioeconomic status and parenting status – a lot more can be done through policy formulation, public education and advocacy to reduce the risk other factors poses for crown fracture in children.

One major study limitation was the inability to assess the tooth status radiographically thereby missing out on some cases of crown fractures and other forms of TDI in the study population. Also, being a secondary data analysis, the study did not collect details on the possible causes of crown fracture making our postulation about improving the school environment to reduce crown fracture an unsubstantiated hypothesis for this study. Within the limitation of the study design that a secondary data analysis pose, we were however able to generate evidence on the association between the socioeconomic status of the child, the parenting status and crown trauma for the study environment; a data that had not been generated before now.

## Conclusions

The study found a low prevalence of TDI in this study population. Significant risk indicators for crown fracture were being a male, living with single parents and having high or low socioeconomic status. Unfortunately, these are biological variables that are difficult to ameliorate. It would be important to identify other environmental associated risk indicators for crown trauma in the study environment, such as the home and school related environment factors that can be ameliorated to reduce the risk for crown trauma.

## Abbreviations

SES: Socioeconomic status
TDIs: Traumatic dental injuries
